# Integrated care for children living with complex care needs: an evolutionary concept analysis

**DOI:** 10.1007/s00431-023-04851-2

**Published:** 2023-02-13

**Authors:** Lorna Cassidy, Mary Brigid Quirke, Denise Alexander, Jo Greene, Katie Hill, Michael Connolly, Maria Brenner

**Affiliations:** School of Nursing, Midwifery and Health Systems, University College Dublin, Health Sciences Building, Belfield, Dublin 4 Ireland

**Keywords:** Integrated care, Children with complex care needs, Concept analysis, Integrated care programme, Child

## Abstract

Children with complex care needs (CCNs) are in need of improved access to healthcare services, communication, and support from healthcare professionals to ensure high-quality care is delivered to meet their needs. Integrated care is viewed as a key component of care delivery for children with CCNs, as it promotes the integration of healthcare systems to provide family and child-centred care across the entire health spectrum. There are many definitions and frameworks that support integrated care, but there is a lack of conceptual clarity around the term. Furthermore, it is often unclear how integrated care can be delivered to children with CCNs, therefore reinforcing the need for further clarification on how to define integrated care. An evolutionary concept analysis was conducted to clarify how integrated care for children with CCNs is defined within current literature. We found that integrated care for children with CCNs refers to highly specialised individualised care within or across services, that is co-produced by interdisciplinary teams, families, and children, supported by digital health technologies.

*  Conclusion*: Given the variation in terms of study design, outcomes, and patient populations this paper highlights the need for further research into methods to measure integrated care.

**Table:** 

**What is Known:**
• *Children with complex care needs require long-term care, and are in need of improved services, communication, and information from healthcare professionals to provide them with the ongoing support they need to manage their condition*.
• *Integrated care is a key component in healthcare delivery for children with complex care needs as it has the potential to improve access to family-centred care across the entire health spectrum*.
**What is New:**
• *Integrated care for children with CCNs refers to highly specialised individualised care within or across services, that is co-produced by interdisciplinary teams, families, and children, supported by digital health technologies*.
• *There is a need for the development of measurement tools to effectively assess integrated care within practice*.

## Introduction

Children with complex care needs (CCNs) have a dynamic and individual set of care needs that can only be addressed through long-term and ongoing support delivered by a range of different health and social care providers across diverse sectors [1]. They often live with multiple concurrent conditions with significant functional limitations, high symptom burden, along with frequent and prolonged hospital stays, and in some cases high rates of pediatric emergency department (ED) and pediatric intensive care unit (PICU) admissions [1–3]. It has been cited internationally that children with CCNs and their families experience difficulties accessing adequate and consistent psychological support, health education, and community services [2, 4, 5]. Dissatisfaction with care coordination and inadequate communication among multiple providers involved in the child’s care are also two key issues experienced by families, which leads to the provision of inconsistent information and confusion for families over points of accessing care [1, 2, 6]. A key barrier in accessing the relevant support and information families require is the fragmented design of healthcare systems, as many operate using traditional models of care that are designed to deliver episodic care, and do not adequately support children and families who require ongoing care [3].

Integrated care is a term that has been frequently linked to children with CCNs and is increasingly viewed as a critical component of their care delivery [2, 7]. There are a range of different models and frameworks that have been developed to support the integration of care; however, many are not targeted specifically towards the needs of children with CCNs. Integrated care for children with CCNs focuses on the provision of physical, psychological, and social care with a central focus on the needs of children, young people, and families [8, 9]. There are many definitions of integrated care, which can be understood differently. Fundamentally however, as a concept, integrated care must encompass two key principles. Firstly, key aspects in the design and delivery of care systems that are fragmented must be combined ‘to integrate’ to form a whole, and secondly, the concept must deliver ‘care’ through providing assistance or treatment to people in need. To deliver integrated care, healthcare systems need to provide child and family centred care across the entire health spectrum [8, 9]. This can only be achieved through integrated service delivery, whereby professionals from different sectors collaborate and communicate closely in a flexible and team-oriented way to ensure that shared goals of care are delivered [3, 8, 10].

The increase in published literature describing the various definitions, models, and frameworks that support integrated care shows that our understanding of integrated care has evolved. Nevertheless, there are no universal standard practice guidelines for healthcare professionals (HCPs) to follow to assist with implementation [8]. This article builds on previous research conducted by members of the research team that highlighted how the needs of children with CCNs cannot be addressed by one profession or discipline, and that integration of health services along with a strong multidisciplinary approach is critical to ensure high-quality competent care is provided for this population [6]. The purpose of this paper is therefore to provide clarification on the concept ‘integrated care’ through systematically analysing how it is defined within current scientific literature in relation to integrated care programmes delivered to children with CCNs. An evolutionary concept analysis was selected as the most appropriate methodology as it supports the view that concepts constantly evolve and change over time and are influenced by the context within which they are used [11–13].

## Methods

**Fig. 1:**
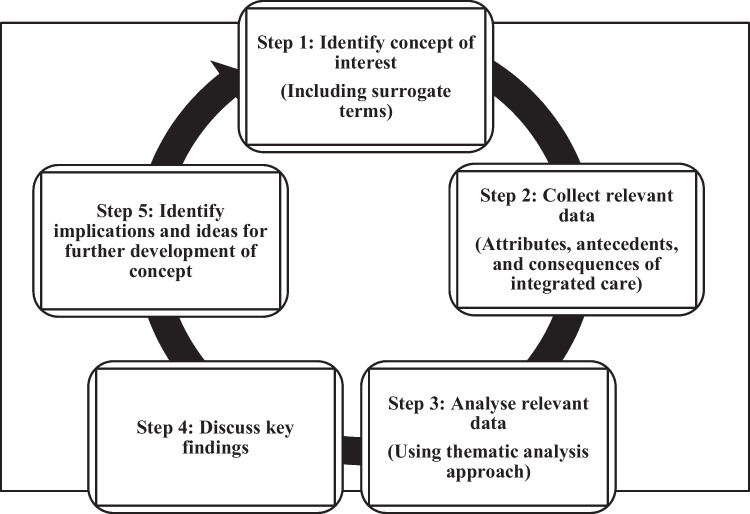
Concept analysis steps guided by Rodgers' evolutionary methodology [12]

Rodgers’ evolutionary methodology was used to clarify and analyse the concept of ‘integrated care’ provided specifically to children with CCNs [12]. This methodology was well suited to analyse the concept of ‘integrated care’ as the definition and application varies within scientific literature, and there is a need for a shared understanding to help inform the design and implementation of integrated care programmes within clinical practice. Scientific literature was reviewed, and core steps were followed (Fig. 1). An inductive form of analysis was used whereby data was gathered and analysed from each study using a thematic analysis approach to identify commonalities to help establish the key attributes of the concept and its features [12].

### Data sources and search strategy

The main concept of interest at the outset was ‘integrated care’; however, this was considered too broad as the intention of this analysis was to explore integrated care delivered to children with CCNs in a healthcare context; therefore, the concept ‘integrated care’ was explored through the lens of integrated care programmes in healthcare. A robust systematic search was conducted using a three-strand approach, and databases searched for relevant articles were as follows: CINAHL, Embase, Medline, PsycINFO, and Web of Science. A scoping search was run in CINAHL, Embase, Medline, and PsycINFO to identify control language and MESH headings, which created a list of synonyms. A further scoping search was then carried out to identify key words related to the following concepts: *children, complex care needs, and integrated care programme*. The final search consisted of a combination of key words and MESH terms that were applied to all databases using the “AND” and “OR” operators. Searches were limited to articles published over the last 12 years (2011–2022), in English language, and had to include the term ‘integrated’ in the title or abstract. Snowballing was also used to review the reference lists of resulting articles to identify potentially relevant articles. All articles were imported into Covidence with all duplicates removed, and the remaining abstracts and full-text articles were screened for eligibility.

### Data extraction and analysis

**Fig. 2:**
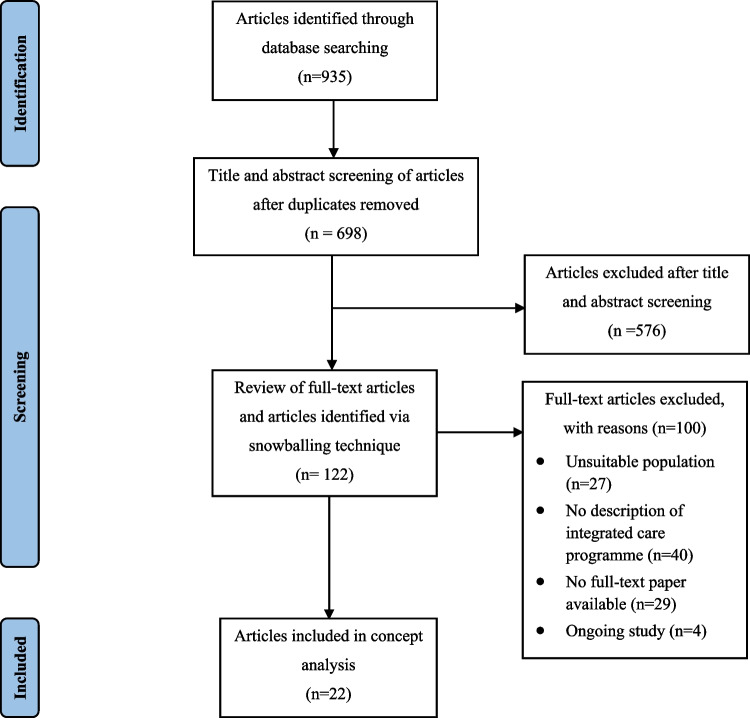
Data selection process [29]

**Fig. 3:**
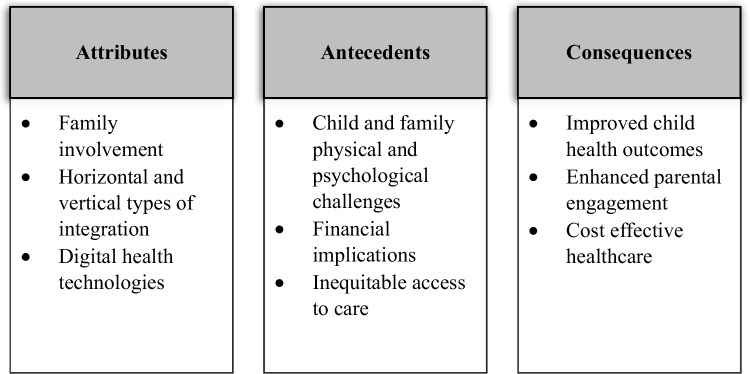
Attributes, antecedents, and consequences of integrated care programmes

A total of 937 articles were retrieved, 935 were found via database searching (Embase *n* = 487, Medline *n* = 195, CINAHL *n* = 125, PsycINFO *n* = 64, Web of Science *n* = 64), and by the means of the snowballing technique, two additional articles were found. Two hundred and thirty-nine duplicates were removed resulting in 698 that were screened for eligibility. Title and abstract screening were combined into one step, resulting in 576 articles that were excluded, with a further 100 full-text articles excluded that did not meet the inclusion criteria. Main reasons for exclusions included no description of the integrated care programme provided (*n* = 40) and no full-text paper available (*n* = 29). A total of 22 articles were included for final analysis (Fig. 2). All included articles were analysed thematically, and data was extracted in relation to the (1) attributes, (2) antecedents, and (3) consequences, as recommended by Rodgers [12] (Fig. 3). Each article was reviewed and read thoroughly by two reviewers, paying attention to key information related to the attributes, antecedents, and consequences of integrated care. Information was entered into a data extraction form developed using Microsoft Word, and a table was used to capture data relevant to three categories (Table 1). The table was then exported into NVivo, where data was analysed and reorganised using a thematic approach guided by Braun and Clarke’s model (2006) [14] to identify common patterns among all studies related to the attributes, antecedents, and consequences.

**Table 1:** Data extraction table

**Number**	**Question**
1.	What are the key attributes of integrated care programmes for children with complex care needs?
2.	What factors (antecedents) are proposed to precede integrated care programmes?
3.	What are the consequences of integrated care programmes?

## Findings

### Surrogate terms

Rodgers outlines that the first step in analysing a concept is to identify surrogate terms that may be used as an alternative to the main concept [12, 13]. Out of 23 studies, only three used the terminology ‘integrated care programme’ [3, 5, 22], and surrogate terms included ‘integrated service’ [15–18], ‘integrated method’ [19], and ‘integrated support system’ [19] (see Table 2). Thirteen articles referred to using a specific integrated model of care [3, 15, 17, 18, 21–29]. Models of care described were chosen specifically to suit the study population and included ‘integrated behavioural model’ [15, 17], ‘family integrated care model’ [24–26], ‘integrated clinic model’ [27], ‘integrated practice model’ [23], ‘integrated complex care model’ [3], ‘integrated care model’ [22, 28], ‘Children’s Treatment Network’[18], ‘The Paediatric Perioperative Surgical Home model’ [21], and ‘collaborative practice model’ [29]. The variation in the terminology highlights how the focus of integrated care has developed and changed over time. From 2012 to 2016, the focus was more on the process of integrating services and hospital departments with very little mention of the concept ‘integrated care’, whereas from 2017 onwards the term ‘integrated care’ appears more frequently, and providing integrated care is a main aim of the majority of studies. Additionally, the term ‘family integrated care (FICare)’ appears to be a more recent term that was only mentioned in studies published within the last three years showing an increase in family-centred care. Furthermore, from 2018 to 2022, specific models of care were mentioned more frequently and used to inform the development of integrated programmes and or services, showing an increase in theoretically driven studies.

**Table 2:** Overview of included studies

**Authors**	**Country**	**Study design**	**Patient population**	**Description of programme**	**Type of integration**	**Terminology**
1. Altman et al. [3]	Australia	Quality improvement project	Children with medical complexity (no sample given, as results will be published in greater detail elsewhere)	The Kids Guided Personalised Services (GPS): linking tertiary and local hospitals for children with medical complexity. The main foundation of the programme was care coordination, 24–7 telephone support provided to families along with a smartphone app [The My Health Memory (MHM)] that directly uploaded child’s electronic medical records and shared the care plan. Families of children frequently attending the emergency department (ED) were encouraged to ring the hotline first for guidance on what service their child should attend	Vertical	Integrated complex care model
2. Black et al. [27]	USA	Quality improvement project	Children with sickle cell disease (SCD) and suspected pulmonary comorbidities (asthma) (2–18 yrs, *n* = 24)	Integrated paediatric SCD and pulmonary care clinic: paediatric haematologist and pulmonologist delivered care in parallel with standard SCD treatments. Interdisciplinary team (paediatric haematologist, paediatric pulmonologist, asthma educator, SCD nurse/clinic coordinator) delivered care. Patients received standard SCD treatment but were also screened for pulmonary symptoms using asthma control questionnaires, along with asthma education and written action plans provided to patients and families	Horizontal	Integrated clinic model
3. Casher et al. [15]	USA	Evaluation study	Patients in paediatric EDs and urgent care (3–19 yrs, *n* = 213)	Enhanced integrated behavioural health service: doctoral-level psychology resident was integrated into the primary team to be the first point of contact for psychiatric consult questions. They reviewed patient medical records and were available for brief consultations. Integrated care consultant reviewed patients’ medical course, conducted brief clinical assessments, and delivered brief psychotherapy or psychoeducation, and co-ordinated with follow-up providers	Horizontal	Integrated service
4. Cohen et al. [23]	Canada	Evaluation study	Children with complex chronic condition that is associated with medical fragility (up to 16 yrs old, children and their primary caregivers, *n* = 81)	Community-based complex care clinic integrated with a tertiary care centre: clinics were staffed by local community paediatricians together with a tertiary care affiliated nurse practitioner. They were run as a co-management model with existing primary care providers. Patients were referred by their primary care providers. Clinics were run weekly at each site *(2 sites: two community general hospitals- Hospital for Sick Children, Brampton Civic Hospital)*. The nurse practitioner participated via telemedicine during periods of bad weather. Visits were focused on care coordination, complex symptom management, and goal setting. A care plan was developed by the nurse in partnership with the family for all patients, using an electronic template with info on goals of care, patient-specific emergency management guidelines, updated medication lists, and contact details for the medical team. Care plan was given to families and uploaded to an e-portal so it was available to providers across the continuum. Health support was also available from a social worker and dietician when needed, and other community providers. Communication by the family through email and telephone with the nurse was encouraged	Vertical	Integrated practice model
5. Corrigan et al. [25]	USA	Parallel group randomized trial	Mother-infant dyads (preterm infants in NICU scheduled for post-retinopathy of prematurity (ROP) examination, *n* = 100)	Family-integrated care in the neonatal intensive care unit (NICU): Mothers participated in two separate music therapy interventions (recorded maternal singing and heartbeat for her infant post ROP examination and recorded infant heartbeat with mother-preferred music for the mother’s personal use). Music therapists met with mothers separately, and sessions began with assessing the mother’s overall coping with hospitalisation. The infant’s heartbeat was recorded, and mothers picked a song meaningful to them to be paired with the heartbeat. Music was provided to each mother in the MT group digitally or on a CD. They were advised to use the recordings when they wanted	Horizontal	Family integrated care
6. Dahhan et al. [31]	Netherlands	Feasibility study	Ethnic minority children with chronic diseases (*n* = 189)	The Mosaic Outpatient Clinic (MOC): involved support offered by a trained team of professionals: 2 supervising consultant paediatricians, 4 student healthcare workers as cultural mediators. Trained student healthcare workers performed consults (45 min) with patients to explore culturally sensitive issues, and parents were asked to specify what healthcare problems they experienced with their children and how the care could be optimised. Patients were seen 3–4 times in the MOC before they were referred back with a report to their own paediatrician at the general POPD. An individually tailored plan was proposed by the healthcare worker and finalised with input from paediatricians and discussed with patients and parents together	Vertical	Integrated clinic
7.Graham et al. [5]	America	Feasibility study	Children with respiratory technology dependence (30 days–22 yrs, *n* = 320)	The Critical Care, Anaesthesia, and Perioperative Extension (CAPE) Program: aimed to provide comprehensive service through individually tailored care with home visits, liaising with inpatient services, rehabilitation programs and outpatient clinics, school programs, and community services. A key feature was the 24–7 family driven access to critical physicians and other professionals. A key objective was to partner with community providers for routine health maintenance while addressing gaps in care related to the child’s underlying complex condition and needs. The CAPE program was provided in lieu of a traditional, hospital-based pulmonary/respiratory clinic program. Patients received scheduled home and clinic visits at regular intervals with unrestricted family program utilisation. Included MDT including nurse practitioner and social worker	Vertical	Integrated care programme
8. Grimes et al. [29]	America	Pilot study	Children referred by paediatricians for outpatient child psychiatry evaluation (4–19 yrs, *n* = 32)	Integrated care for children via a collaborative-practice model (CPM): CPM aimed to foster interdisciplinary collaboration between mental health and primary care. To facilitate integration child mental health specialists joined paediatrics team meetings and psychiatry notes were shared with paediatricians via the electronic health record (EHR). Child psychiatry and family support specialist (FSS) staff were available weekly in the paediatric clinics. FSS staff were non- clinician parents with lived experience of caring for a child with mental or substance use disorder needs. FSS staff brought a family perspective to interactions with clinicians and translated families concerns to the clinical teams. Used parent interviews, follow-up and home visits. The intervention was individualised for each child on the basis of team-identified needs and level of treatment intensity, and youth and family preferences	Vertical	Integrated care
9. Guarnaccia et al. [35]	Italy	Implementation study	Children with asthma (6–15 yrs, *n* = 260)	Integrated clinical and educational pathway for asthmatic children and adolescents (IOEASMA): included three visits with an 8-week interval. Following the first visit, an individual asthma education course was offered to all enrolled patients. The three clinical visits were followed by two visits 6 months apart. The first assessment included family history, past medical history (PMH) and history of present illness (HPI). During the pathway, prick-test and spirometry were performed. Once the control of the disease was evaluated, daily therapy was introduced or modified. At the second, third and two follow-up visits, symptoms were monitored, disease control was evaluated and daily therapy was adjusted as directed by guidelines	Horizontal	Integrated pathway
10. Hall et al. [28]	America	Pilot study	Infants in PICU (aged 3–72 months, *n* = 33)	Paediatric Critical Care Neurotrauma Recovery Program (PCCNRP): consisted of paired visits with physicians and neuropsychologists from evaluation to referral for targeted intervention. Participants were cared for in the PICU with a neurologic illness or injury and then attended the initial post-discharge follow-up appointment. All children received a physical and neurological examination from the paediatric critical care physician. All children had a brief neurodevelopmental assessment with the neuropsychologist, with parent/caregiver interview and the completion of parent-reported outcome measures. Evaluations were completed in real-time and feedback provided to families with both providers present. The length of the appointment was 2 h	Horizontal	Integrated care
11. Harden et al. [16]	UK	Quality improvement project	Paediatric kidney transplant recipients (15–18 yrs *n* = 12)	Integrated paediatric-adult clinical service for patients with kidney failure: joint medical clinics at paediatric centres with a team of a paediatric nephrologist and paediatric renal transplant nurse specialist working jointly with an adult nephrologist and adult renal transplant nurse. The joint clinics occurred every 4 months and saw patients jointly by the two teams with transfer to the adult clinic occurring by the age of 18. At each of the 3-h joint medical clinics held at the paediatric centre, 4–5 patients were seen, with individual MDT consultations up to 45 min long. Patients were seen alone to promote autonomy but also with family members to discuss progress and future management. Before transfer to the adult clinic, the patient meets with a youth worker, with at least one community visit, to look around the adult clinic informally	Horizontal	Integrated service
12. Husted et al. [19]	Denmark	RCT	Adolescents with type 1 diabetes (13–18 yrs old, *n* = 71)	Guided self-determination- Youth (GSD-Y) intervention: Intervention group: used guided self-determination (GSD), a life skills approach to facilitate empowerment in the patient-provider relationship. Two paediatric physicians, five paediatric diabetes nurses, and two dieticians provided the GDS-Y intervention as part of their conventional outpatient clinical care. Intervention consisted of 8 sessions over an 8–12-month period with each session lasting 1 h. Involved 18 semi-structured reflection sheets for adolescents, 5 for parents, and 6 if the adolescent was visiting a dietician. Adolescents were also invited to attend one session without their parents to facilitate conversation about personal affairs Treatment-as-usual group: Adolescents in the control group were also offered 8 sessions, which were scheduled equal to the intervention group across an 8 to 12-month period. They also received typical outpatient care	Horizontal	Integrated method
13. Leahy et al. [21]	USA	Implementation study	Patients undergoing congenital cleft repair (*n* = 235)	The Perioperative Surgical Home (PSH) model- a patient-centred integrative model of delivering healthcare during the entire patient surgical/procedural experience, from the decision of surgery through the recovery phase. A paediatric PSH was developed for patients with a diagnosis of laryngeal cleft undergoing endoscopic surgical repair. An interdisciplinary team (anaesthesiologists, surgeons, nurses, quality improvement specialists, IT personnel, finance professionals) met regularly during the design, development, and monitoring phase. Duplication of care and areas of high resource use were identified and redesigned through the integrative approach. Key caregivers were brought together for clinical decision making, input, and evaluation	Horizontal	Integrated care coordination pathway
14. Lee et al. [37]	Hong Kong	Retrospective study	Paediatric patients who underwent sclerotherapy (*n* = 49)	Joint Vascular Anomalies Clinic: MDT delivers one-stop integrated care to patients with low-flow vascular malformations via a comprehensive team of specialists and nurses. Patients are assessed at the clinic which is held every 2 weeks. Patients are concurrently assessed by the paediatric surgeon and interventional radiologist, with a detailed clinical history, physical examination, and physical measurements taken	Horizontal	Integrated care
15. McLean et al. [24]	Canada	Cluster RCT	Infants born at < 33 weeks of gestation and receiving minimal or no respiratory support and their parents (*n* = 126)	Family Integrated Care (FICare): Intervention group: parents at FICare sites committed to spending > 6 h/day at the infant’s bedside. Parents received enhanced training in the day-to-day care of their infant including bathing, feeding, providing skin-to-skin care, dressing, administering oral medications, taking temperature, and interacting with their infant to support development. Parents were encouraged to participate with the medical team on medical rounds, clinical decisions about their infant’s care, and chart their infant’s growth. Informal peer-to-peer support was also provided and parent and social work involvement in the education sessions, parents were provided with psychosocial support Control group: received standard care	Horizontal	Family integrated care approach
16. Menon et al. [20]	USA	Pilot study	Patients admitted with or developed acute kidney injury (AKI) in all non-ICU inpatient units (6 months–18 yrs, *n* = 225)	Clinical decision support (CDS) system (combining electronic alert and standardised care pathway (SCP)) for children with AKI): integrated new SCP within the existing paging system used in hospitals to improve detection and management of AKI through clinical decision support (CDS) systems. AKI e-alerts were sent to the primary in-house provider (intern, senior resident, nurse) who acted as the clinical contact for the patient. A secure paging system was used to send a short system-based text-page with details of patient and stage of AKI	Vertical	Integrated clinical support system
17. Pereira et al. [17]	USA	Evaluation study	Patients referred for psychiatric consultations (*n* = 363)	Integrated care continuity clinic: embedded three predoctoral paediatric psychology residents (PSY) into paediatric medical resident continuity care clinics in an urban academic medical centre. Each PSY resident was embedded within three half-day resident primary care continuity clinics (4-h clinics held in the morning and afternoon). Paediatric resident continuity clinic teams were made up of two to four paediatric or medicine/paediatric residents, one PSY resident and one onsite licenced psychologist. PSY residents were involved in patient care during clinics in a number of ways: targeted diagnostic assessment, brief interventions, and collaborative treatment planning. A key component of the learning environment was follow-up discussion with the collaborating paediatrician provider. Discussions typically occurred on the same day as the intervention to allow for team collaboration on a joint treatment plan for the patient. If additional psychotherapy was needed, PSY residents could continue to work with families in their psychology outpatient clinics or could refer them to community resources	Horizontal	Integrated service
18.Pratt et al., [22]	USA	Longitudinal design	Youths attending a weight-treatment clinic and their caregivers (8–18 yrs, *n* = 267)	The Paediatric Healthy Weight Research and Treatment Center (PHWRTC): provides a comprehensive multidisciplinary intervention to youth referred by their primary providers due to a concern about the youth’s weight and risk of weight-related comorbidities. The care team uses an integrated care model, and care is co-ordinated between medical and mental health providers, with shared care treatment plans. Providers include two physicians that rotate clinic time, a dietician, one family therapist, and a family therapy intern. At each visit, the patients and their caregivers met with the physician and mental health provider together and a dietician. Height, weight, BMI, and blood pressure are tracked by the medical provider at each visit, and depression is tracked by the family therapist. Regular follow-up appointments are made typically every three months	Horizontal	Integrated care model
19. Stelwagen et al. [26]	Netherlands	Qualitative study	Mothers (*n* = 27) and fathers (*n* = 9) of newborns who were hospitalised for at least 7 days	FICare: integrated maternity and neonatal care to keep parents close together in an integrated maternity and level 2 neonatal ward. Provided a combination of different care models known to promote parent empowerment. Conducted in a mother and child centre to stimulate parent empowerment by integrating the concepts of single-family rooms, couplet care and FICare (family integrated care). Integrated nursing team consisted of specialised maternity nurses, specialised neonatal nurses, and specialised mother and newborn nurses. Care was able to be provided by one single nurse. Keeping parents and new-borns together was supported by offering room service and facilities for parents	Horizontal	Family integrated care
20. Tom et al. [32]	USA	Evaluation study	Parents of children with chronic diseases (*n* = 256)	Integrated personal health record (PHR): electronic personal health records (PHR) linked to the patient’s electronic medical record. Allowed patients to view immunisations, lab results, health plan visits, to manage their condition. The aim of providing this to parents of children with chronic conditions was to improve overall care experience by increasing their understanding of their child’s illness	Horizontal	Integrated personal health record
21. Waters et al. [36]	Australia	Implementation study	Children with anxiety symptoms (4–18 yrs, *n* = 243)	The Take Action Program: utilised a cognitive behavioural intervention (CBI) with anxious children and youth. It was delivered in a classroom-based format and consisted of eight weekly 1-h sessions. Intervention included: psychoeducation about anxiety, training in relaxation techniques, identifying anxious self-talk, development of strength and problem-solving skills and social skills. Children were given psychoeducational handouts after each session to give to their parents, to keep parents informed or what their children were learning and provide practical parenting strategies. Delivered by psychologists during school and class time	Vertical	Partnership that integrates school-based, evidence-informed treatment delivery with clinical education
22. Ye et al. [18]	Canada	RCT	Children with special healthcare needs (CSHCN) (0–19 yrs, *n* = 445)	Children’s Treatment Network (CTN): CTN group: each child was assigned a service navigator who assessed the child’s health conditions, then a trained service coordinator followed up with the family and working with the family an individual team of service providers was formed to suit the child’s specific health and social needs. This integrative team worked with the family to form a single plan of care for the child, and the service coordinator organised the delivery of services in accordance with the plan. The team met regularly with parents for ongoing revisions and assessment of the plan. All assessments and notes concerning the child were shared in an electronic system by all team members Control group: families continued to manage services for their child in a self-directed manner	Vertical	Integrated service

### Attributes

Attributes are known as clusters of characteristics that are categorised based on their frequent occurrence within the literature to help define a concept [11, 12]. The identified attributes of integrated care include family involvement, horizontal and vertical types of integration, and digital health technologies.

### Family involvement

The manner in which families are involved in an integrated care programme was a defining attribute in the literature reviewed, as it was viewed essential that family members were included as part of the treating team. In the majority of studies, this was achieved through the establishment of shared care or treatment plans with close input from the family and medical team [17, 18, 22, 23, 28–30]. There was a strong focus on promoting parental autonomy and empowerment by allowing parents to contribute towards the decision-making processes regarding their child’s condition [3, 24, 27, 32], and in some cases, parents were given permission to attend medical rounds alongside the healthcare team [24]. Typically, families were involved in every step of developing shared care plans, and collaboration between family and HCPs was commonly used to set goals of care to meet their child’s needs. In four studies, education was key to enhancing family involvement, with educational sessions or written information provided to family members and children with advice and information on how to manage their care needs [24, 25, 27, 32]. Furthermore, giving the family access to unrestricted and 24–7 specialist advice and guidance from HCPs when at home was considered an integral part of an integrated care programme [5].

### Horizontal and vertical types of integration

While specific types of integration were not explicitly mentioned or defined in the literature reviewed, each study was categorised based on the types of integration defined by the World Health Organisation [34]. Integration can be described as horizontal when organisations or services that deliver care at a similar level come together, and vertical when organisations or services delivering care at different levels are brought together [33, 34]. The most dominant type of integration that emerged from the literature was horizontal (*n* = 14), and the remaining studies used vertical integration (*n* = 8). Horizontal integration was the most popular type of integration used which could suggest this is less difficult to implement as it focuses on combining healthcare services that operate on a similar level. Studies that used horizontal integration delivered highly specialised and coordinated care by integrating existing departments and services based within hospital settings. An example of this included psychological support that was provided within paediatric outpatient departments and EDs, whereby psychology and behavioural experts were included as part of the healthcare team. Psychological support was provided during or alongside routine clinic appointments for children who were described as high risk for developing psychological sequalae including children with obesity, type 1 diabetes, and asthma [17, 19, 22, 35].

Behavioural and supportive interventions were also integrated into existing practice, for example, a music therapy intervention was incorporated into neonatal units [25], and a therapeutic-educational intervention into asthma clinics [35]. Integrated ‘joint-care clinics’ were commonly referred to and involved the integration of staff from two existing hospital departments to provide integrated care for the child at their visit. Departments that delivered integrated care through joint care clinics included maternity and neonatal [26], renal [16], critical care and neurotrauma [28], and pulmonology and haematology [27]. Children were seen by multiple specialists during their appointments or concurrently to address other health care needs and screen for common comorbidities related to their specific condition. Studies also used vertical integration, which involved care that was delivered by a range of professionals that provided different levels of care based in different healthcare settings, such as tertiary and primary care, and community services. Providers from different levels of care worked together as a team to deliver individualised care to children. Examples included care of children with respiratory technology dependency [5], anxiety disorders [37], medical complexity [3, 23], chronic diseases [31], special health care needs [18], acute kidney injury [20], and children referred for psychiatric evaluations [29], in place of traditional hospital-based clinic programs.

### Digital health technologies

Digital health technologies were commonly used to facilitate communication and sharing of medical information between family members and members of the healthcare team, and within healthcare teams. The literature describes electronic alerts as mainly being used in the form of paging systems and patient portals [18, 20, 23, 29, 32]. A smart-phone app was also used to share information and updates to families about their child’s health status, and to make medical notes available to providers across the continuum [3]. One integrated care clinic also used telemedicine, to ensure that family members were able to attend the clinic virtually if they were not able to attend in-person [23]. Sharing medical information with family members and giving them access to their child’s medical records via digital technology were considered important in all papers as it gave family access to information conveniently and quickly.

### Antecedents

Antecedents are understood as incidents or phenomena that occur before the development of a concept [12, 13]. The antecedents to integrated care primarily stem from challenges associated with the multitude of appointments and services required for the child and family. Antecedents were identified as child and family physical and psychological challenges, financial implications, and inequitable access to care.

### Child and family physical and psychological challenges

A strong body of evidence shows that the lack of integration within healthcare systems results in decreased quality of life for children with CCNs [3, 24, 25, 27]. There is considerable disconnect described in the literature between services for children within the community and across different disciplines, and a lack of flexibility which can result in discontinued care [15, 23, 37]. Children with complex conditions often live with multiple comorbidities, some of which are not treated or screened for during their routine appointments. Waters et al. (2015) and Cohen et al. (2012) both describe a need to provide children with appointments that address multiple health conditions at once to reduce their physical burden. Furthermore, the lack of consistency in treatment, discontinuity of healthcare and long-term support for children with CCNs have a direct impact on the child and family’s psychological health. Children with CCNs often suffer with lifelong symptoms and have an increased risk of developing psychological conditions [28, 29]. Their conditions may affect their social and personal lives, with many children missing school due to appointments or symptom burden, limiting their level of engagement with their peers. Evidence has also found high rates of non-adherence to medications and disengagement with treatment as a result [19, 35]. The lack of adequate and accessible services for children with CCNs who experience poor mental health reinforces the need for integrated care programmes that combine services to address psychological and physical health symptoms [28, 29].

The psychological impact of caring for a child with CCNs and navigating the necessary but disparate healthcare systems further supports the development of integrated care programmes. Attending multiple hospital appointments and travelling to different centres to access treatment can be disruptive, and emotionally draining for families, as they adjust their lives to cope with the demands of care [17, 18, 23]. This was especially evident among parents caring for infants in neonatal ICU facilities or those requiring long-term mechanical ventilation as they experience isolation and separation from their children for long periods of time [5, 24].

### Financial implications

The financial impact of caring for a child with CCNs can be described as a burden that both parents and healthcare systems experience. The literature highlights how parents often must travel to different treatment centres and clinics to seek out the best specialised care for their child, which can result in time off work, reduced work hours, and in some cases job loss [18, 23, 32]. The high level of care required for children with CCNs results in increased rates of health care utilisation. They require more hospital resources, experience prolonged hospital stays, and frequent ED and PICU admissions, which creates further financial strain on healthcare systems [3, 20, 23, 24, 26, 31]. Evidence described in the literature has indicated that an integrated care approach could be cost-effective, as it has the potential to reduce ED admissions and hospital resource usage [3, 5, 17].

### Inequitable access to care

Unequal access to healthcare was another key issue described frequently, and it was highlighted that access to care can be dependent on socioeconomic and geographical factors. This was most evident among studies that were conducted in  the United States, Canada, Australia, and the Netherlands. Healthcare systems were also described as lacking cultural competency in the care of children from ethnic minority backgrounds, leading to disparities in healthcare provision, and decreased quality of care [29, 31]. Geographical limitations were mentioned as another barrier in accessing services, as families based in rural locations often do not have the same access to services and may struggle to cope with the financial implications of travelling to urban areas for multiple appointments and specialist treatments [17, 18, 23]. Lack of services within the community and close to home was another issue frequently mentioned [23, 27, 36].

### Consequences

The final stage in analysis was the identification of consequences, which are the result of the use of the concept in practical situations [12, 13]. The consequences identified reflect the predominantly positive outcomes of integrated care in relation to the child, parents, and healthcare systems. The consequences therefore are improved child health outcomes (physical and psychological), enhanced parental engagement, and cost-effective healthcare.

### Improved child health outcomes (physical and psychological)

A range of positive physical health outcomes for children were found within the included studies, such as improved quality of life [23], reductions in body mass index (BMI) scores [22], resolution of symptoms [37], reductions in asthma presentations [29], improved recovery rates in preterm infants who underwent eye exams [25], improved asthma control [35], and reductions in unplanned transfusions [27]. There were also high rates of engagement found with improvements in attendance at follow-up appointments [16, 29], management and adherence to medications [16, 20], and motivation to self-manage symptoms [19]. Furthermore, there were also improvements in relation to child psychological health, with studies finding lower levels of depression [22], anxiety [36], and suicidal thoughts [17]. Only two studies reported negative outcomes, which included no improvement in diabetes management [19], or psychological quality of life [18].

### Enhanced parental engagement

Improvements in parental outcomes were also found, including decreased maternal stress levels [24] and high satisfaction rates of integrated care programmes [5, 31]. It was also found that parents felt respected by HCPs as they had the opportunity to gain more autonomy when it came to the care of their child, as they felt more involved and part of the healthcare team [24–26]. In terms of negative results, one study found that parents did not have enough time to engage with an integrated electronic paging system [32], and another study found no improvements in mother-infant bonding [25].

### Cost-effective healthcare

Financial benefits were also highlighted, with parents of children with CCNs experiencing a reduction in travel expenses [22] and travel time [29]. Integrated care programmes were also described as cost-effective for healthcare systems as results found a decrease in the number of ED admissions [17, 29, 31, 35], a decrease in no-shows for appointments [31], an increase in the availability of ICU beds [21], and less expenses used on pharmacological therapies to treat symptoms of asthma [35]. Two studies described how, encouraged by the results, integrated care clinics were continued on a temporary basis as there was no additional space or efforts required [27, 31].

## Discussion

This paper highlights the lack of conceptual clarity in the literature regarding integrated care programmes for children with CCNs. The term ‘integrated care’ was used infrequently within the included literature; however, studies that have used the term have increased over the last five years. Furthermore, the increase in integrated models of care highlights how programmes and services are being developed in a more robust and theoretically driven way to suit specific patient populations.

Integrated care as both a concept and a process has been incorporated into adult services to ensure a more unified approach to health and social care services. The overall aim being to deliver care in a safe, and efficient manner as close to the person’s home as is possible. Essentially integrated care means changing how care is provided to promote more independent and healthier lives. A particular focus for the organisation of integrated care is the perspective of the patient who, although living with complex care needs, also needs to be supported to avoid further illness and to be empowered to be involved in their own care decisions. This can only be achieved when multidisciplinary and cross service planning and delivery is coordinated and collaborative. As a concept, integrated care is not new in the context of the care of adults with complex care needs. As populations age and individuals are living longer, the need to address the consequences of a fractured health and social care system are paramount so that the holistic needs of individuals can be fully addressed.

The term, however, has not yet been widely adopted when considering children with CCNs, which does reflect how ill-defined the concept is for this population, as the success of a concept is dependent on how frequently it is used within a given field [38, 39]. If the usage of a term or concept appears to be inconsistent, it may be beneficial to modify the definition to reflect how the term is used within broader literature [38]. Therefore, based on the defining attributes and evidence from the literature reviewed, we propose *that integrated care for children with CCNs refers to highly specialised individualised care within or across services, that is co-produced by interdisciplinary teams, families, and children, supported by digital health technologies.*

A key attribute of integrated care was the utilisation of digital health technologies, which in recent years has been viewed as a critical tool in healthcare [9, 10, 33]. The COVID-19 pandemic created a major global healthcare crisis that shifted the focus of healthcare delivery to a digital approach, as HCPs embraced technology to provide remote monitoring and management of patient care [3, 40]. Evidence has found that families of children with CCNs have reported high levels of satisfaction when it comes to digital health technologies in particular virtual clinics and video consultations, as they involve less time and travel, two key logistical barriers frequently mentioned by families preventing them from accessing adequate services [3, 41, 42]. Digital health technologies contribute to the effective delivery of integrated care, through facilitating efficient communication and sharing of information, and information technology (IT) has the potential to greatly improve access to this [43]. Digital health has reorganised the delivery of healthcare systems around the needs of patients, with new types of services emerging such as electronic health records (EHR) that support integrated care [40, 44, 45]. EHRs have improved the availability and accessibility of healthcare data, by allowing for data to be shared among multiple healthcare systems and teams and making it more accessible for families and patients. This is further supported by the findings of this paper, as sharing medical information with family members was viewed as a crucial element of an integrated care programme, as it made them feel respected by HCPs and more autonomous [24–26].

A key finding of this paper was the number of studies published within the last 5 years focused on improving access to psychological and physical healthcare services for children with CCNs. The lack of adequate and accessible services for children who experience poor mental health is a rising concern, and something that is mentioned frequently within the literature. In many countries, mental and physical healthcare continues to be delivered in silos, with mental health services often separated and not a part of hospital settings [28, 29, 46]. There is pressure on paediatricians to deliver psychological support to children with CCNs, as there is a high prevalence of psychological sequalae among this population; however, this is often challenging to deliver due to lack of training and time constraints, leading to HCPs feeling ill-equipped to deal with mental health issues [15, 29, 46]. A number of included studies integrated psychological support into paediatric outpatient and EDs, and a range of positive outcomes were found including reduced anxiety and depression [22, 36], and increased motivation to self-manage symptoms [19]. Although the studies were conducted in different countries within different healthcare systems, the results suggest that optimising the use of a combination of pharmacological and non-pharmacological strategies is recognised as beneficial to improve the overall health of children with CCNs [46].

The findings of this paper indicate that studies focused on providing integrated care to children with CCNs are increasing over time; however, further research is warranted and there is a need to develop tools that can help facilitate integrated care and ensure a patient-centred approach. The development of integrated care programmes could be strengthened by including tools such as care mapping, which allows patients to express and visualise what integrated care means and looks like to them [47].

## Limitations

This paper found a lack of published studies that reported negative results and it is possible that publication bias may have occurred as a result, as only two included studies reported negative outcomes [18, 19]. Additionally, a large proportion of articles were excluded due to the unavailability of full-text papers (*n* = 29), and despite all corresponding authors being contacted for further information, a small number of authors replied with the majority explaining that they had no intention of publishing their results. This is concerning as healthcare research aims to translate research into practice, and without published results, it is difficult to understand and implement programmes [48]. HCPs play a vital role in conducting healthcare research, especially when it comes to evaluating integrated care programmes; however, they experience barriers that make it difficult for them to publish results such as lack of time, resources, and funding [49, 50]. This reinforces the need for improved funding that can support HCPs who are involved in clinical research to disseminate their results and share their knowledge to inform practice.

## Conclusion

There has been a significant growth over the past decade in literature to support the development of integrated care for children with CCNs, but little guidance on what processes or elements are essential for successful implementation of integrated care programmes. This paper draws on key elements as demonstrated in the literature that are commonly included within an integrated care programme. We identified that integrated care for children with CCNs *refers to highly specialised individualised care within or across services, that is co-produced by interdisciplinary teams, families, and children, supported by digital health technologies.* However, there was wide variation in terms of outcomes, study design, and patient populations, which suggests that there is no ‘one size fits all’ approach that can be taken. Further research is needed to identify measurement tools that can be implemented across healthcare systems to assess integrated care; however, it is hoped that the findings of this paper can provide direction for future related work.

## Data Availability

The data used in this study is available in Table 2 in the article.

## Abbreviations

BMI: Body mass index
CCNs: Children with complex care needs
COVID-19: Coronavirus disease 2019
ED: Emergency department
EHR: Electronic health record
HCPs: Healthcare professionals
IT: Information technology
PICU: Pediatric intensive care unit
QI: Quality improvement
